# Changes in Dietary Patterns and Clinical Health Outcomes in Different Countries during the SARS-CoV-2 Pandemic

**DOI:** 10.3390/nu13103612

**Published:** 2021-10-15

**Authors:** Roxana Filip, Liliana Anchidin-Norocel, Roxana Gheorghita, Wesley K. Savage, Mihai Dimian

**Affiliations:** 1 Faculty of Medicine and Biological Sciences, Stefan cel Mare University of Suceava, 720229 Suceava, Romania; roxana_filip@yahoo.com (R.F.); roxana.puscaselu@usm.ro (R.G.); wesley.savage@usm.ro (W.K.S.); 2 Regional County Emergency Hospital, 720224 Suceava, Romania; 3 Integrated Center for Research, Development and Innovation in Advanced Materials, Nanotechnologies, and Distributed Systems for Fabrication and Control, Stefan cel Mare University of Suceava, 720229 Suceava, Romania; dimian@usm.ro; 4 Department of Computers, Electronics and Automation, Stefan cel Mare University of Suceava, 720229 Suceava, Romania

**Keywords:** pandemic, prevention, food hygiene, bioactive compounds, diet, lifestyle

## Abstract

Coronavirus disease 2019 (COVID-19), caused by severe acute respiratory syndrome coronavirus 2 (SARS-CoV-2), has led to an excess in community mortality across the globe. We review recent evidence on the clinical pathology of COVID-19, comorbidity factors, immune response to SARS-CoV-2 infection, and factors influencing infection outcomes. The latter specifically includes diet and lifestyle factors during pandemic restrictions. We also cover the possibility of SARS-CoV-2 transmission through food products and the food chain, as well as virus persistence on different surfaces and in different environmental conditions, which were major public concerns during the initial days of the pandemic, but have since waned in public attention. We discuss useful measures to avoid the risk of SARS-CoV-2 spread through food, and approaches that may reduce the risk of contamination with the highly contagious virus. While hygienic protocols are required in food supply sectors, cleaning, disinfection, avoidance of cross-contamination across food categories, and foodstuffs at different stages of the manufacturing process are still particularly relevant because the virus persists at length on inert materials such as food packaging. Moreover, personal hygiene (frequent washing and disinfection), wearing gloves, and proper use of masks, clothes, and footwear dedicated to maintaining hygiene, provide on-site protections for food sector employees as well as supply chain intermediates and consumers. Finally, we emphasize the importance of following a healthy diet and maintaining a lifestyle that promotes physical well-being and supports healthy immune system function, especially when government movement restrictions (“lockdowns”) are implemented.

## 1. Introduction

Since early 2020, the novel severe acute respiratory syndrome coronavirus 2 (SARS-CoV-2) causing the COVID-19 pandemic has led to health care systems around the world facing insufficient resources [1]. As new SARS-CoV-2 variants arise, individual immunodeficiencies and co-morbidity factors, diet and lifestyle factors, vaccine effectiveness, and vaccination rates will determine infection rates and persistence of the pandemic, and as we have seen with the delta variant, health care systems everywhere struggle to treat the number of severe COVID-19 cases, in many situations because of compromised immune systems due to poor diets and lifestyle habits. The appearance of COVID-19 revealed shortcomings with the capacity for public health systems to respond early to a novel pathogen and prevent a global pandemic, and moreover, it highlighted an emerging public health crisis of comorbidities due to diet and lifestyles that exacerbate COVID-19 progression and outcome. While scientific research and public health systems need to adapt quickly to combat emerging pathogens, as we see in the current pandemic where medical science produced the fastest development of a vaccine that has and continues to prevent countless COVID-19 cases requiring hospitalization and reduced deaths due to co-morbidities, it also became abundantly clear that the general state of public health can determine the outcome of infection and the magnitude of excess mortality [2,3].

Despite efforts to contain the spread of SARS-CoV-2, this new, contagious virus is challenging social welfare, public health, and the medical and scientific research community through its unpredictable spread via new variants, long-term effects of infection (i.e., “long covid” and organ injury), and differing outcomes across demographic groups. Initial measures were focused on containing the spread of SARS-CoV-2, through promoting mask-wearing, hand-sanitizing, and stay-at-home measures, all of which are similar to measures implemented to slow the Spanish flu pandemic in 1917–1918 [4]. However, these are only measures of containment and do little to address the problem of infection outcomes, which are exacerbated by the modern diet, nutrition, and lifestyle behaviors, all of which have been linked to prolonged infection, admission to intensive care units, and excess death rates usually linked to co-morbidity factors.

We know from previous outbreaks of viruses similar to SARS-CoV-2 that higher pathogenicity is generally correlated with lower transmissibility, and vice versa. As far as we know now, SARS-CoV-2 has lower pathogenicity and moderate transmissibility compared to MERS-CoV, avian SARS-CoV-1, Ebola, and H7N9 [2]. However, in the many areas of the world that lack sufficient medical resources to treat severe covid cases, infections are resulting in greater mortality due to COVID-19 than in areas with higher vaccination rates and greater availability of health care. These outcomes may be mitigated by dietary strategies aimed at maximizing dietary approaches that benefit infection outcomes and minimizing lifestyle behaviors that contribute to excess mortality. Further, as in the early stages of the pandemic, the World Health Organization (WHO) produced guidelines on water, sanitation, hygiene (WASH), and waste management approaches relevant to viruses, including coronaviruses [5]. While global vaccination and boosters are the main axes of containing the virus, and thereby mitigating transmission rates, delays in access to and deployment of the vaccine in parts of the world will ensure that SARS-CoV-2 variants arise via mutations, and continue to spread across populations. This means other measures remain crucial to minimizing viral spread; as we saw earlier in the pandemic before vaccination rollouts, public health measures, such as focus on using proper hygiene, social distancing, mask use, and population lockdowns, had the effect of slowing down the pandemic, but not without physical, social, and mental health consequences. This had unforeseen consequences for population health, namely that people may not have had access to healthy foods and instead were faced with limited choices that affected what they consumed, how often, and their ability to exercise or adopt positive lifestyle behaviors.

How SARS-CoV-2 presented itself to the human populations is still not known, and will likely be difficult to track because of rapidly evolving genomes (i.e., mutation), genetic recombination (as yet unknown), and switching from reservoir to novel host species. It took more than a decade after the SARS-CoV-1 outbreak in China in 2002 before it was reported that this coronavirus originated in bats and spread to palm civets, then infecting humans [6,7]. In the case of the MERS coronavirus outbreak, a clear transmission route is not exactly known, but it is theorized to also have originated in bats, with camels perhaps the intermediate host leading to transmission to humans [8]. To date, no wild animal tested in the region of the outbreak (i.e., “ground zero”) in Wuhan, China has been identified as transmitting the SARS-CoV-2 virus to humans, but given that bats are known reservoirs for SARS-like coronaviruses, a natural origin is plausible, and likely involves contact with an intermediate host as in the case for MERS and SARS-CoV-1 [8,9]. The salient point of this is that we will likely continue to host SARS-CoV-2 in the human population, and aside from the impossible task of chasing spike protein mutations with designer vaccines, our best chance at reducing the impact of it is through population-level hygiene, food chain hygiene, and diet and healthy lifestyle awareness.

The SARS-CoV-2 virus reached pandemic proportions in short time due to modifications in the receptor binding domain (RBD) that enhances viral binding to the angiotensin converting enzyme 2 (ACE2) receptors, with particular affinity to pulmonary tissues (pneumocytes) where the virus can have more severe disease outcomes [10]. More concerning is that ACE2 is also highly expressed on adipose cells, which suggests that weight gain via fat deposition (through unhealthy dietary habits) can pose a serious problem with COVID-19 recovery by being a viral reservoir in overweight people. This may explain some of the evidence that obesity is a significant co-morbidity factor with COVID-19 hospitalization and mortality, and further evidence that dietary habits during the pandemic may continue to exacerbate severe disease outcomes. Transmission can also happen directly from the reservoir host to humans without receptor-binding domain adaptations [11]. The bat coronavirus that is currently in circulation possesses spike proteins that facilitate human infection. Interspecies transmission from animals to humans is possible by the high plasticity in receptor binding and the possibility of viral antigenic “make-up” by mutation and recombination. The in vitro and in vivo studies of isolated virus demonstrate that there is a potential risk for the re-emergence of coronavirus infection from viruses that are currently circulating in bat populations in nature [12]. Given these realities, and the fact that vaccines are chasing a moving target, the host immune response to combat infection has re-emerged as an important focus on preventative health.

Recent published articles address changes in food hygiene, lifestyle, and diet from different regions of the world during the COVID-19 pandemic, but given the recent emergence of SARS-CoV-2, changes are ongoing and much more data are being gathered. Ultimately, understanding how the pandemic is affecting mental, social, and physical health in the global human population relative to pre-pandemic conditions is important for defining the broader health impacts of COVID-19 beyond direct clinical disease pathology [13,14,15,16].

A healthy diet, based on plant-healthy fats and proteins, together with regular exercise and sunlight exposure, is of paramount importance to help prevent viral infection by strengthening the immune system. However, sedentarism, unease, and tediousness caused by social isolation could lead to changes and worsening of lifestyle patterns while also promoting binge-eating, which is worsened by limited access to healthy food rich in natural vitamins, minerals, and antioxidants [17].

The aim of this review is to highlight the latest evidence on how the clinical pathology of COVID-19, including comorbidities and immune response due to lifestyle behaviors and diet, may exacerbate outcomes and prolong the severity of the novel SARS-CoV-2 pandemic.

## 2. Clinical Pathology of SARS-CoV-2 Infection and COVID-19 Syndrome

Severe acute respiratory syndrome (SARS-CoV-2) causes COVID-19, a disease that presents a complex of syndromes, including severe specific contagious pneumonia (SSCP) and Wuhan pneumonia [18]. While this coronavirus has less severe pathogenesis compared to SARS-CoV-1, it is highly transmissible, demonstrated repeatedly by the rapidly and continuously increasing number of COVID-19 cases since it emerged in December 2019. The mean incubation time of SARS-CoV-2 in familial clusters is reportedly 3 to 6 days [19,20]. Similar to MERS and SARS-CoV-1, the severity of COVID-19 is higher in age groups above 50 years [21,22]. Since the onset, while the per-capita mortality rate is lower than recorded in outbreaks of SARS-CoV-1 and MERS, the high rate of transmission means that specific demographics are particularly impacted by COVID-19 [23]. Information obtained from outbreaks in Thailand, South Korea, China, and Japan confirm that COVID-19 patients usually had mild manifestations compared to those with SARS-CoV-1 and MERS, and with a much larger sample size owing to the high rate of transmission. Regardless of the SARS-CoV type, the primary barrier against SARS-CoV-2 is the immune system, and the first line of defense is mast cells in the submucosa of the respiratory tract and nasal cavity [24].

Severe interstitial inflammation of the lungs is caused by invasion of pulmonary parenchyma by SARS-CoV-2 [25]. In radiology, the characteristic image is “ground glass “opacity of the lungs. The lesion initially involves a single lobe but later expands to other lobes [26]. Lung tissue biopsies of COVID-19 patients reveal diffuse alveolar damage, desquamation of pneumocytes, hyaline membrane formation, and cellular fibromyxoid exudates indicative of acute respiratory distress syndrome [27]. Hematological findings show lymphocytopenia, both with and without leukocyte abnormalities, and the degree of lymphocytopenia is positively associated with disease severity [26].

## 3. COVID-19 and Comorbidities

**Figure 1:**
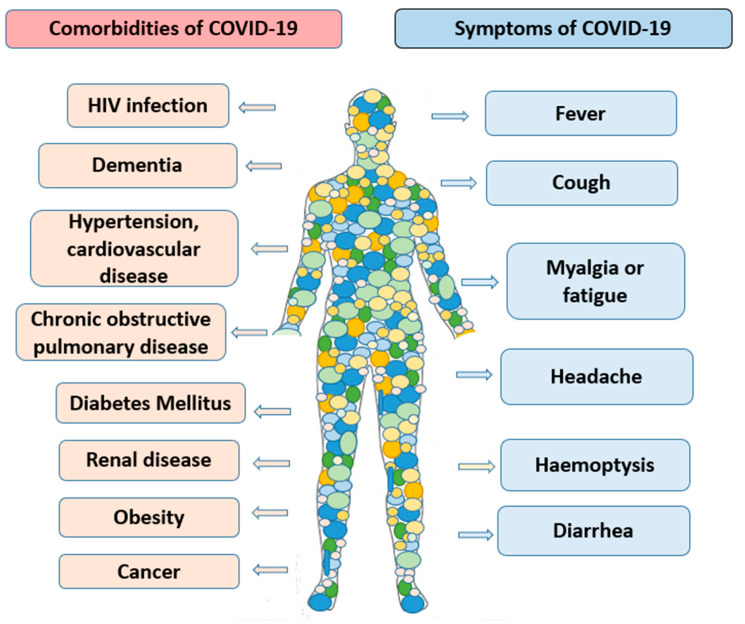
The main comorbidities and symptoms of COVID-19.

Cardiovascular disease, obesity, hypertension, diabetes, and other pre-existing conditions are highly correlated with the severity of COVID-19 infection and cause excess deaths via co-morbidities (Figure 1) [28]. The clinical manifestation and relevance of specific comorbidities due to COVID-19 infection is heterogenous [29]. In a large study of 460 general practices in England, of the 4300 COVID-19 patients with hypertension, about 20% died within 1 month of infection. Of note, the authors did not find any correlation between COVID-19 diagnosis or hospitalization and blood pressure control [30].

Patients with underlying cardiovascular disease and pre-existing blood vessel damage, such as atherosclerosis, may be at higher risk for severe disease. In addition to respiratory infection and inflammation, the SARS-CoV-2 coronavirus can directly and indirectly affect the renal system and cardiovascular tissue, which cause organ and tissue damage to the kidneys, heart, and blood vessels, and exacerbate inflammation that induces cytokine storms.

Similar to increased COVID-19 severity in patients with cardiovascular disease, especially hypertension, many studies show greater severity of infection in diabetics [31,32,33,34]. Current data indicate that diabetes in COVID-19 patients is correlated with a two-fold increase in mortality as well as severity of COVID-19. A meta-analysis of 30 studies and 16,003 patients conducted by Kumar et al. [35] suggests that diabetes and COVID-19 infection are significantly correlated with mortality [36]. The method for influencing the relationship is rigorous glucose monitoring and consideration of drug interactions.

As treatment for COVID-19, there are several pharmaceutical drugs options, such as lopinavir and steroids, which have a risk of hyperglycemia. In contrast, hydroxychloroquine may improve glycemic control in diabetic patients with decompensated refractory treatment [37,38]. It remains uncertain which treatment is suitable and works best for COVID-19 disease, and if treatment of diabetic patients should be different from those without diabetes. It is also uncertain whether specific diabetes drugs, such as DPP4 inhibitors, increase or decrease the susceptibility or severity of coronavirus infection. Isolated reports of new-onset diabetes in COVID-19 cases may suggest that coronavirus infection is directly cytotoxic to pancreatic islet β cells. Careful investigation [39] indicated that interaction of the coronavirus and diabetes is mediated by systemic inflammation and/or metabolic changes in adipose tissue, muscle, or liver, and not by direct infection of pancreatic cells.

Chronic obstructive pulmonary disease (COPD), a complex disease related to airway and/or alveolar abnormalities, is a lung dysfunction that is manifested by limited airflow mainly caused by exposure to harmful gases and particles over a long period of time (e.g., tobacco smoke). A meta-analysis of 15 studies that examined 2400 confirmed COVID-19 cases suggested that patients with COPD were at higher risk of more severe disease outcomes, with 60% higher mortality [40]. Multivariate logistic regression models identifying risk factors for positive SARS-CoV-2 tests in smokers were inconclusive [41]. While it cannot be concluded that smoking enhances SARS-CoV-2 infection, smokers are more likely to present a cough that can signal pulmonary distress and possible advance of COVID-19 infection; however, more testing is required to determine whether this population is directly susceptible to the virus by pulmonary infection, or by suppressed immune system function [42,43,44].

HIV infection serves as a model of cellular immune deficiency and it seems that antiretroviral therapy is thought to have various effects against the new coronavirus [45]. Given the fact that HIV positive patients may be at higher risk from other infectious diseases such as sexually transmitted diseases, these percentages were so low that some experts have already speculated on potential protective factors [46].

There are still debates about the effects of antiretroviral therapy against SARS-CoV-2. Regarding lopinavir, there is currently concrete evidence that it does not work. Regarding tenofovir alafenamide, there are some chemical similarities with remdesivir and it has been shown to bind to SARS-CoV-2 RNA polymerase with high binding affinity, being suggested as a potential treatment for COVID-19 [47]. However, the most serious concern about HIV is the collateral damage induced by COVID-19 [48]. The story of immunosuppression is uncertain, and the available data are insufficient to draw any conclusion. Despite the large lack of data, numerous views and guidelines have been published on how to manage immunosuppressed patients (who may be more susceptible to COVID-19 infection) and the development of severe cases [49,50,51,52,53].

A big challenge of the pandemic is to offer continuous care for cancer patients. Cancer patients are more vulnerable due to their underlying disease and immunosuppressed condition, and may therefore be at increased risk of developing severe complications due to the coronavirus. In fact, COVID-19 triage and management may leave some vital activities uncovered, such as treatment administration or surgery, and also a fragile immune system. It is well known that suboptimal synchronization and delayed oncological treatment can lead to disease progression, leading to reduced survival outcomes. There are various recommendations to minimize the exposure of cancer patients to COVID-19 without compromising the oncological outcome of radiation for breast cancer [54], hematopoietic cell transplant [55], and leukemia treatment [56].4. Immune Response to SARS-CoV-2.

In the ongoing SARS-CoV-2 pandemic, the consequences of infection range from asymptomatic to mild to moderate symptoms in most affected COVID-19 cases, but also can have a rapid and progressive disease that damages organs and leads to early deaths, in some cases as soon as 14–21 days from onset of infection. Since the start of the pandemic, facts have been complicated surrounding whether the virus can continue to be transmitted by asymptomatic individuals [57,58], and certainly by those with upper respiratory tract symptoms, or interstitial pneumonia that can progress rapidly to respiratory failure and acute respiratory distress syndrome, in which mechanical ventilation and admission to an intensive care unit and culminating in multiorgan failure [59,60,61]. Disease spread is correlated with longer viral shedding periods, encountered especially in asymptomatic patients [62]. After viral contamination, an effective adaptive immune response able to neutralize new antigens can be expected to develop in 14–21 days [63].

Antiviral innate immunity consists of coagulant factor, and components of the complement and fibrinolytic systems, soluble proteins that recognize glycans on cell surfaces, interferons, chemokines, and naturally occurring antibodies (mainly IgM but also IgA and IgG). The cellular components are natural killer cells and other innate lymphoid cells but also gamma delta T cells, which generally limit the spread of viral infection [64]. The viral spike protein preferentially binds to the angiotensin converting enzyme 2 receptor (ACE-2), prevalent in cells in the mammalian respiratory tract. Glycosylation of the viral surface can affect some aspects of virus biology, such as cell tropism, stability of protein components, camouflage of recognized antigens by neutralizing antibodies, and recognition by immune mechanisms.

Antiglycan antibodies are naturally identified in serum, i.e., they are identified in the absence of previous immunization, similar to natural ABO antibodies. Like ABO antibodies, they belong to the IgM class. Natural IgM concentrations appear to reflect some of the clinical severity patterns in COVID-19 [65]; they decrease significantly with age (>40 years) and are found in lower concentrations in people with blood type A. A protective role of high anti-A antibody titers described for SARS-CoV-1 [66] has been suggested for the SARS-CoV-2 [67].

Mannose binding lectin (MBL) is one of the components of the complement system in innate immunity, which recognizes mannose residues in the membrane of a variety of microorganisms, and acts as a soluble pattern recognition receptor (PRR). This recognition component activates the complement system, induces inflammation, and improves phagocytosis. MBL can bind to coronavirus, conducting to C4 deposition in the virus and in experimental models, decreases the capacity for infection [68]. The existence of mannose-rich glycans in the S1 region of SARS-CoV-2 has led to the hypothesis that glycan recognition and binding to MBL may inhibit the S1–ACE2 interaction [69]. However, with age, serum MBL levels decrease [70].

The first line of the innate immune response against viral infections is represented by type I interferons. They induce viral resistance in both infected cells and neighboring cells by interfering with cellular and viral replication. In MERS and SARS-CoV-1 infection, delayed production of interferon I favors the accumulation of inflammatory monocyte–macrophages [62,71].

The key diet-related changes in the developmental process of disease progression in humans include increased production of reactive oxygen species, oxidative stress, development of hyperinsulinemia, insulin resistance, low-grade inflammation, and an abnormal activation of the sympathetic nervous system and the renin–angiotensin system. Further, diet plays an important role in epigenetic changes and fetal programming that may have large effects on immune system efficiency. This suggested pathomechanism also explains the close relationship between obesity and the wide range of comorbidities, such as type 2 diabetes mellitus, cardiovascular disease, etc., and diseases of similar etiopathology. Changing lifestyle behaviors in accordance with human genetic makeup, including diet and physical activity, may help prevent or limit the development of these diseases [72]. COVID-19 poses a serious challenge to health-care systems worldwide, with an enormous impact on health conditions and loss of life at a remarkable scale. Notably, obesity and related comorbidities are strictly related with worse clinical outcomes of COVID-19 disease. Recently, there is a growing interest in the clinical use of ketogenic diets, particularly in the context of severe obesity with related metabolic complications that are ameliorated through ketogenesis. Ketogenic diets have proven effective for a rapid reduction in fat mass, preserving lean mass and providing an adequate nutritional status. In particular, the physiological increase in plasma levels of ketone bodies exerts important anti-inflammatory and immunomodulating effects, which may prevent infection and potential adverse outcomes of COVID-19 disease [73].

### 3.1. Nutrients and Food Bioactive Components Involved in Immune System Stimulation

The immune system is one of the most important defense mechanism of body against disease, and the survival of humans is dependent on this system of fighting against viruses or pathogenic microorganisms [74]. There are studies that have indicated that some nutrients can have effects on immune functions through cell activation and modification of both production of signaling molecules and gene expression. Several micronutrients, such as vitamins and minerals, have essential roles in both adaptive and innate immune responses, and micronutrient homeostasis is central to the maintenance of a healthy immune system (Table 1). The efficacy of micronutrients in infections can be influenced by different factors, such as the dose, duration of administration, type of pathogen, genetics, age, lifestyle, and nutritional and immunological status [75].

There are studies that emphasize a significant relationship between immunity, diet, and disease susceptibility. Nutritional deficits in macro- and micro-nutrients can affect the immune system and resistance to infection. Various functional food plants, such as pepper, garlic, turmeric, and onion, may have immunomodulatory and antiviral properties (Table 2) [75].

### 3.2. The Effects of Isolation, Quarantine, and Lockdowns on Dietary Health

Measures taken by governments around the world to contain the spread of COVID-19 have had measurable impacts on health and habits of people everywhere. A general consequence of quarantine is a change in lifestyle: reduced physical activity and unhealthy dietary choices (Table 3) [91]. Access to healthy foods, such as fresh produce, has been limited, and people have been in lockdowns which prevent outdoor movement and access to sunlight and clean air. Those who have acquired the disease or have been quarantined have often been deprived of sources of higher-nutrition foods [92]. In addition, containment measures restricting free movement and creating physical lockdowns have been detrimental to healthy lifestyles. Regulations aimed at containing viral spread had differing outcomes on different demographics groups, communities, and socioeconomic groups. In particular, failures in infrastructures and supply chain resilience during the early and mid-stages of the pandemic disrupted food availability. For less economically fortunate populations, the breakdown in supply chains resulted in limited access to fresh produce, and instead forced a switch to processed foods that have a longer shelf life. Notably, the connections for supply chain deliveries to communities that are further away from distribution centers/ports/hubs/farms, or that would otherwise depend on imports for healthier foods, had reduced availability of healthy food options. This exacerbated public health issues that have been being largely overlooked by media and governments throughout the pandemic. In contrast, in more economically well-to-do communities, fresh imports and local produce options were available due, in part, to demand, economic liquidity of the communities, and proximity to distribution hubs in those areas. It is well known that a diet rich in fresh produce and whole foods is necessary for healthy immune function, and is thus preferable to a high-calorie processed food diet that increases the risk of developing and even aggravating autoimmune problems and chronic diseases [93]. Recent work reported by Fernández-Quintela et al. in 2020 [94] found that two particular omega-3 fatty acids, eicosapentaenoic acid (EPA) and docosahexaenoic acid (DHA), are both effective at inactivating entrapped SARS-CoV-2 viruses by modulating optimal lipid conditions to reduce viral replication. Both EPA and DHA can also inhibit cyclooxygenase enzymes (COX), which inhibits prostaglandin production, reducing tissue inflammation. Quarantine isolation and lockdown interventions for COVID-19 created, especially for older adults, a severe sense of social isolation and loneliness with potentially serious mental and physical health consequences. The impact was disproportionately amplified in those with pre-existing mental illness, who often suffered from loneliness and social isolation prior to the enhanced distancing from others imposed by the COVID-19 pandemic public health measures. Older adults are also more vulnerable to social isolation and loneliness as they are functionally very dependent on family members or support by community services [95].

#### 3.2.1. Spain

A recent survey of 1036 individuals in Spain reported that, during the pandemic, people consumed more fresh produce as well as fish than before [96]. Another study of 1073 persons reported decreased consumption of poultry and mammal, as well as rice and pasta [97]. Further, a larger study of 7514 individuals reported that people generally consumed a Mediterranean diet more than they did before the onset of the pandemic [98]. These results suggest that people in Spain sought foods that are part of a healthy diet and lifestyle, which may reduce the incidence of severe COVID-19 outcomes.

#### 3.2.2. Italy

Italy was one of the first countries most affected by COVID-19, when hospitalization cases rapidly overwhelmed public healthcare capacity. Early in the pandemic the public faced lockdowns, and that coupled with panic, led to fresh food shortages. In contrast to findings from populations in Spain, a study of 1519 people in Italy reported that the diet of the average person increased in the consumption of frozen foods and foods made with refined sugars [99]. Grant et al., 2021, collected data from 2678 people and observed that many improved the quality of their diet by increasing the consumption of fruits, vegetables, legumes, nuts, fish, or shellfish. However, unfavorable dietary changes were also reported; people consumed an excess of sweets, pastries, and comfort foods than they reportedly did before the pandemic [100]. In another study, 3533 individuals reported a decreased intake of fresh fish, processed sugar foods and baked goods, food delivery, alcohol intake, and an increase in homemade recipes (pizza, sweets, and bread), vegetables, cereals, white meat, and hot beverages consumption [101]. Increased alcohol consumption was observed in a study of 1383 participants, who, according to their responses, also chose foods high in carbohydrates, such as potatoes, cereals, fruits, leading to weight gain amongst the respondents; conversely, this same group reduced their consumption of dairy products, vegetables, and red meat. Anxiety, fear, stress, or moments of boredom have encouraged over 40% of people in Italy to eat foods high in refined processed sugars and oils, leading to weight gain and possible side effects related to COVID-19 infection and disease severity [102]. However, these variations in the weekly frequency of food consumption did not alter the adherence score to the Mediterranean diet, which remained at medium-high values [103]. In this sense, Italians have been trained to transform their green spaces into food gardens, especially taking into account the benefits of eating fresh fruits and vegetables. The television programs followed the training of small farmers, the purpose being not only to obtain their own crops, but also to focus their attention on recreational activities to maintain mental health [104].

#### 3.2.3. France

A study of 938 individuals showed increased intake of fresh produce, legumes, and seafood. Consumption of refined sugars, processed meats, sweet drinks and alcoholic beverages also increased, leading to a decrease in nutritional quality of the average diet [105]. A total of 11,391 participants surveyed in the first 8–13 days after home confinement measures were implemented revealed increased consumption of caloric and salty foods (28.4%), alcohol (24.8%), tobacco (35.6%) and even cannabis (31.2%) [106]. Almost a quarter of French people engaged in behaviors that contributed to poor health outcomes, in many cases by stress management through eating more and unhealthy foods, and lack of physical activity due to confinement indoors [107].

A study of 498 parents of children aged 3.0–12.3 years presented no changes in eating behavior for other reasons than a change in eating habits. A significant decrease was observed for rules and limits around unhealthy foods, setting, and on scheduled meals. Children increased their consumption of high carbohydrate sources and processed foods, including sweets/chocolates, fruit juices, soft drinks, chips/crackers, ice cream, pastries/sweet cakes, dessert cream, milk, yogurt/cheese/quark, fresh and dried fruits, and a significant decrease in the consumption of compote/fruit in syrup [108].

#### 3.2.4. Greece

Unlike other countries heavily affected by COVID-19, such as Spain or Italy, the Greek population was not so emotionally involved, and the signs of anxiety and depression were less obvious among adults. Thus, the emotional eating of unhealthy foods was not so high [109].

Nearly one in three of 2258 participants reported that they changed their dietary habits during the pandemic towards a healthier diet rich in fruits, vegetables, salads, green vegetables, cereals, legumes, and olive oil, and consumed less meat, especially processed meat [110]. Similarly, another study of 741 individuals report a prudent dietary pattern containing of fruits, vegetables, fish, and rice [111].

#### 3.2.5. Denmark

The data presented by Giacalone, et al., 2020, based on a questionnaire with a number of 2462 subjects, suggest that the pandemic affected the lifestyle and eating habits of some adults living in Denmark. The main findings include the fact that they ate more frequently. During this period, survey respondents reported they consumed more processed, canned, frozen, or ready-to-eat food, and reduced consumption of bread, alcohol, and dairy [112]. Moreover, unhealthy eating habits were observed, such as the increased intake of pastries and carbonated beverages [113].

#### 3.2.6. Poland

People in Poland reported positive dietary changes, consuming less red meat, processed flours and baked goods, prepared foods, fast-food, canned meat products, as well as energy drinks and refined sugars. However, increased consumption of alcoholic beverages and sweets, both of which are poor dietary choices, contributed to unhealthy weight gain [114]. Increased BMI was associated with less frequent consumption of vegetables and fruits during quarantine, and higher adherence to meat, dairy, and fast-foods. Increased alcohol consumption was reported in 14.6% of study participants, with a tendency to drink more among regular alcohol consumers [115].

#### 3.2.7. China

In China, the general frequency of intake of fresh vegetables, fruits, soybean products, and dairy decreased during the lockdown. Average weekly consumption of rice decreased, but there were increases by younger age classes in wheat products, other staple foods, fresh vegetables, fresh fruit, preserved vegetables eggs, fish, and dairy products. Furthermore, the frequency of sugar-sweetened beverage consumption had decreased, while the frequency of other beverages had increased [116].

#### 3.2.8. United States

In general, people shifted their diet away from healthy animal proteins, fruits, and vegetables, reportedly due to increased cost because of supply chain issues related to the pandemic. Local supply of food was disrupted, which in turn affected local economies that led to social, mental, and physical health changes. The municipal authorities developed programs to support and protect food security during the pandemic, but especially in the post-pandemic period. In the U.S., people reported experiencing greater stress, anxiety, and boredom, which led to overeating and weight gain. People ate more, and in particular they ate more processed snacks and comfort foods, which are rich in additives and fats, processed trans-fats, high salt, and sugar [117]. According to statistical analysis, the population consumed mainly red and processed meats, fast food, sweets, and refined cereals during the pandemic, and with the return of the US economy, prices fell and facilitated access to vegetables, oils, nuts, and lean proteins [118].

Regarding food hygiene, it is suggested that consumers living in communities with COVID-19 cases have higher food safety knowledge scores, disinfect cooking surfaces more, pay more attention to food safety information, and have more timely access to food safety news. So, people with COVID-19 pandemic-related information tend to have higher food safety knowledge and practice food safety behavior [126,127].

### 3.3. Importance of Lifestyle in Prevention of COVID-19

**Figure 2:**
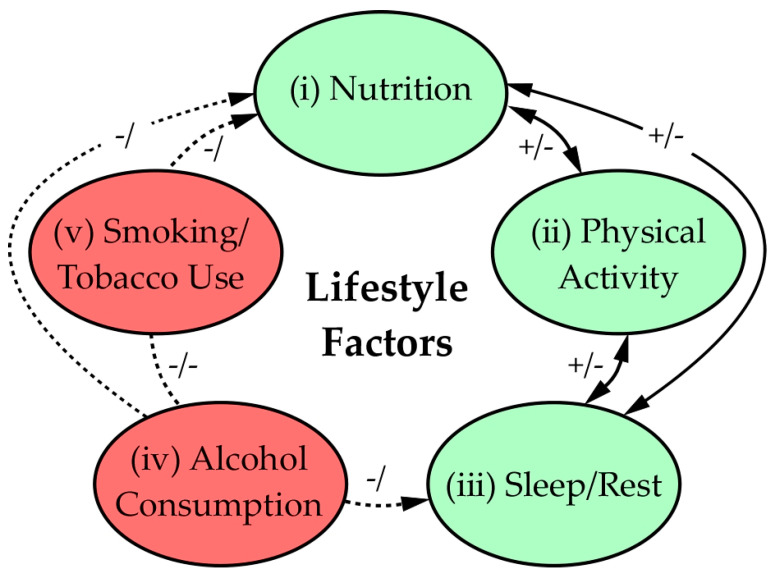
Lifestyle factors that affect clinical health outcomes in COVD-19 infections. Dashed lines indicate negative effects, whereas solid lines connect factors known to improve health outcomes; arrows denote interaction direction(s). Red indicates negative lifestyle factors; green indicates positive factors. (i) Nutrition is essential in supporting immune system function and is affected by other factors positively/negatively (+/−) [129]. Poor quality rest or lack of physical activity can limit the benefit of nutrition as a health factor (−), and the inverse is true (+). (ii) Physical activity and regular exercise help mitigate disease effects and are related to nutrition and rest similarly [130]. (iii) Quality sleep and rest contribute to healthy outcomes, and affect and are affected by nutrition and activity. Note that inadequate sleep can induce stress/anxiety that can exacerbate health outcomes (−). (iv) Alcohol consumption leads to organ stress and dysfunction, depresses the immune system response to viral and bacterial infections, and negatively impacts sleep quality (−/) [131]. (v) Tobacco use has well-known detrimental effects on the immune system and leads to many clinical health problems that exacerbate infectious disease outcomes (−/) [132]. Smoking and alcohol consumption are often correlated and thus have negative interactions (−/−).

Since early in the pandemic, the most effective measures that reduce transmission of SARS-CoV-2 and prevent COVID-19 spread have been physical distancing and proper use of face masks that have multiple layers of tightly woven, breathable fabric, a nose wire, and a thickness that block lights when held up to bright light source [128] (Figure 2).

Lifestyles have changed substantially due to isolation and distancing measures (Figure 2), as people are more sedentary, and the lack of physical activity is correlated with poor dietary changes and unhealthy weight gain, both of which contribute to severity of COVID-19 outcomes. Indeed, obesity and being in poorer health with a less nutritious diet is associated with greater severity of COVID-19 cases requiring hospitalization. Other problematic outcomes of social isolation measures implemented during the pandemic are changes in smoking and sleep habits. Several studies reported associations between sleep disorders and obesity due to increased secretion of pro-inflammatory cytokines by increasing visceral adipose that can contribute to altered sleep-wake rhythms [88,133,134,135].

### 3.4. Food Hygiene in COVID-19 Pandemic

Public health and food safety authorities have found no evidence that SARS-CoV-2 spreads via food [136]. The only transmission path involving food is the packaging, which could be contaminated with coronavirus [137,138,139]. Thus, handling or consumption of contaminated food packaging carries similar risks as other surfaces known to transfer coronavirus [140].

The COVID-19 pandemic has caused temporal food shortages due to supply chains changes, labor shortages [141,142,143,144], training personnel in hygiene, food safety, incident management, recreating business models regarding packaging, and other unanticipated impacts [145]. Furthermore, lockdown measures enacted at regional and national levels, such as the closure of universities, schools, workplaces, restaurants, public events, so-called non-essential businesses, and travel restrictions [146], changed the way people purchased food, where they ate, what they ate, and how their food was prepared [112,147]. Some of these changes may be latent symptoms of post-COVID lifestyles.

The plan for preventing the transmission of the coronavirus includes control requirements for food facility disinfection, sanitation, cleaning, monitoring and screening of workers for COVID-19, education programs to prevent the spread of SARS-CoV-2, and management of sick employees [148].

Inactivation methods (thermal and non-thermal) are effective at minimizing pathogens and viruses in the food sector [149]. For the inactivation of foodborne viruses (e.g., hepatitis A and norovirus) on food matrices or liquids, different thermal treatments have been used [150], such as dry (hot air oven) and humid heat (autoclave) which are very effective methods for inactivating both viruses and bacteria [151,152].

**Figure 3:**
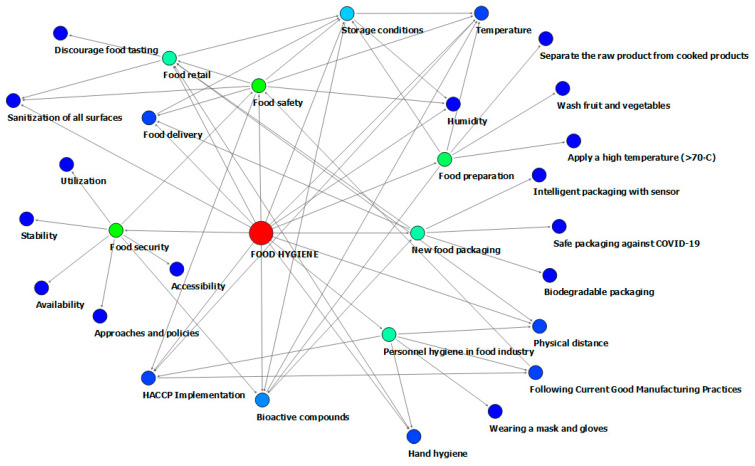
Network connections to food hygiene in food supply systems.

As is specified in research studies, cold-chain food contributes to contamination because coronavirus is stable at 4 °C on poultry, meat, fish, and swine skin, for 3 weeks [137]. Thus, the possibility of transmission through food chain is very high in the frozen food. Therefore, risk management approaches should be adopted to inspect potentially infected foods, especially cold-chain foods (Figure 3) [89].

#### 3.4.1. Food Security

Food security includes availability, accessibility, stability, and utilization of food, at all times for all people. Food must be in sufficient quantities, be safe and nutritious to afford people a healthy life [153].

*Availability*. What foods are available has demonstrably affected the nutritional habits of consumers during the pandemic. The available foods need to be good sources of necessary macro- and micro-nutrients to ensure public health by minimizing severity of COVID-19 cases, and ensuring that basic nutritional requirements are being met is crucial [154].

*Accessibility*. The pandemic has highlighted how much food accessibility can be affected by interruptions in food supply distribution and logistics, leading to rising prices and lack of food options. Restrictions on food logistics are likely to increase transaction costs, and therefore food prices could adversely affect access to healthy food and contribute to food insecurity, obesity, and malnutrition [155]. Reduced access to healthy foods leads to higher consumption of preserved and ultra-processed, which, combined with reduced physical activity, leads to obesity and other diet-related diseases. The ability to deliver whole foods faster and at reasonable costs is a difficult task during the pandemic [156].

*Utilization*. Eating food through a proper diet, drinking water, sanitation, and health care to achieve a state of nutritional well-being in which all physiological needs are met [157].

*Stability*. Food stability emphasizes that all humans should have access to enough food all the time, regardless of any unforeseen risk (such as a pandemic), which could prevent people from accessing food [158,159].

#### 3.4.2. Food Safety in COVID-19 Pandemic

The food industry has Food Safety Management Systems (FSMS) based on the Hazard Analysis and Critical Control Point (HACCP) principles for preventing food contamination and manage food safety risks. FSMSs contain good hygiene practices, zoning of processing areas, storage, supplier control, personnel hygiene, cleaning and sanitation, and fitness to work distribution and transport—all the basic conditions and activities necessary to maintain a hygienic food processing environment [160].

#### 3.4.3. Personnel Hygiene in Food Industry in COVID-19 Pandemic

Cold air conditions in food factories make it particularly difficult to prevent transmission of COVID-19 because the virus is stable over longer periods, and can be moved on aerial particulates by recirculated air systems [161].

Food industry workers are in some cases tested for SARS-CoV-2 to eliminate the potential risk of food contamination [139]. Regular hand washing is crucial in the food sector as well as in all the industries. Similar viruses are spread by droplets when an infected person coughs or sneezes. The WHO recommends measures applicable to the food industry, such as frequent washing of hands with soap and water or alcohol-based disinfectants; maintaining physical distance; and avoiding contact of the hands with the eyes, nose, and mouth. In addition to these practices, the mobility of food industry staff, such as air transport, should be monitored [143,162].

#### 3.4.4. Food Retail in COVID-19 Pandemic

COVID-19 has significantly changed the retail customer experience through changes in availability and increases in pricing. People have to visit more retailers to find specific items, and are often unable to find the types of food and beverages they need to maintain a healthy diet, which can have negative impacts on emotional and mental well-being [163].

Retail workers are often at risk of exposure due to the nature of the workplace that involves interactions with unknown individuals, and consequently we have seen SARS-CoV-2 cluster infections in retail environments and other settings where there is higher traffic of retail individuals that means less control over containment [164].

Measures taken during the pandemic include physical distancing, providing sanitation stations to clean shared shopping equipment, and by regulating the number of customers inside the premises. Physical barriers, such as plexiglass, were deployed to separate food from any risk of danger and to protect staff at cashier point [140].

Food preparation includes measures such as separating the raw product from the cooked product to prevent cross-contamination, as well as washing, rinsing, and sanitizing surfaces or utensils in contact with food and beverage equipment after use. At the same time, practicing good hand hygiene before eating and washing fruits and vegetables with drinking water before consumption are crucial. For cooking food, it is recommended to apply a high temperature (>70 °C) [165].

#### 3.4.5. Food Delivery Include

Customers are more interested in delivery hygiene and food safety with the COVID-19 pandemic because they could be infected with COVID-19 if they contact contaminated food and infected delivery personnel [166]. Contactless delivery in many countries is practiced through the “leave delivery at the door” option or workers leave materials two meters away for customers.

It is also recommended to use face masks and gloves as well as keep physical distance. Employees receiving and delivering must wash or disinfect their hands and implement appropriate hygiene and hygiene protocols. Another measure to avoid contamination is to use an electronic wallet or credit card payment method. It is also recommended that consumers throw away the packaging as soon as possible and wash their hands immediately afterwards [167].

#### 3.4.6. Importance of Smart Packaging with Antiviral Properties

Due to the sensitivity of food security during the pandemic, and faced with the state of global panic on the means of transmission of the virus, the food industry, throughout the world, has been obliged, to also have emergency plans in place. The results of the field survey made it clear that consumers are fearful of the health impacts of COVID-19 on their lives, and may want packaging to be safe, sustainable, and meet their expectations [168].

COVID-19 has had a major impact on consumer choice and eating habits. A review article suggests that during the COVID-19 pandemic, consumers and policy makers responded to an increased perception of the risk to food safety by increasing their dependence on disposable plastic packaging. Attitudes towards food packaging have major implications for both food and environmental policy, thus feeding the need for smart, biodegradable, and safe packaging [169].

**Figure 4:**
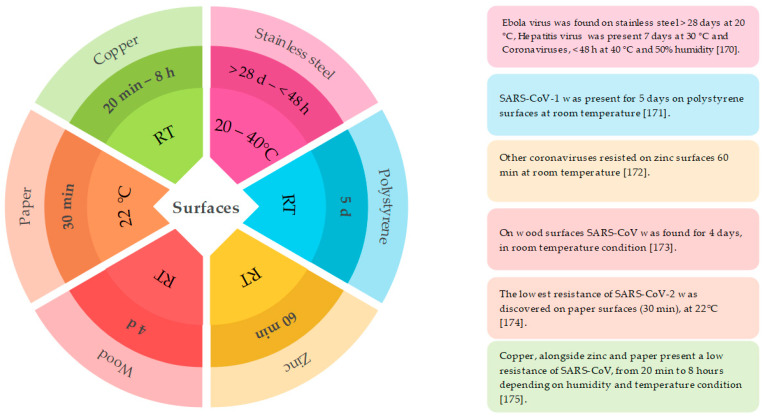
Capacity of SARS-CoV-2 and other viruses to persist on the package surfaces. RT, room temperature: 20–26 °C [170,171,172,173,174,175].

The stability of coronavirus on food packaging has led to the development of materials based on biopolymers with antiviral properties (Figure 4). The use of these materials has shown high efficacy against human norovirus and hepatitis A virus. Some research studies show that the release of ions from the surfaces of copper or copper alloy can help inactivate HuCoV-229E.

The development of biopolymers with antiviral properties and their applications in the food packaging industry remains an open field of research. Recently, it has been reported that the use of nanomaterial-based packaging or films containing zinc, copper, and silver nanoparticles can inhibit SARS-CoV-2, prevent contamination of food packaging surfaces, and thus diminish its transmission [138,176].

## 4. Conclusions

Social limits and movement restrictions introduced on the public during the pandemic have had unforeseen health outcomes that reinforce the importance of maintaining a healthy lifestyle through diet, exercise, and stress management. Because the COVID-19 pandemic changed how and when people could access provisions, what kinds of provisions, and how much were available at any one time, diets in many populations and demographic groups became less healthy compared to pre-pandemic life, notably that people consumed more calories of lower nutritional quality, which can exacerbate COVID-19 outcomes. While not all populations experienced an unhealthy diet shift, it is concerning because, since the start of the pandemic, there have been telling signs that diet patterns led to higher BMI, which, along with obesity, are known to worsen outcomes from COVID-19, with more severe infection requiring intervention via hospitalization, intensive care, and possibly a ventilator, or lead to a fatal outcome. In some populations, the severity of COVID-19 infection seems to be moderated by the consumption of specific micro- and macronutrients, such as those found in the Mediterranean diet. However, an unfortunate outcome of the pandemic has been that, during lockdowns, the slide in adherence to healthy behaviors (i.e., healthy eating, restful sleep, stress management, physical activity, avoidance of risky substances such as smoking and alcohol, and healthy relationships) which can prevent, treat, and even reverse disease, may have been overlooked.

Practicing food hygiene and a healthy diet containing fresh vegetables and fruits are key components of a healthy lifestyle essential for maintaining a properly functioning and efficient immune system to defend against infection and disease. While there is no evidence that the coronavirus can spread directly via foods, packages can be contaminated with SARS-CoV-2, and could transmit the virus; therefore, there may be a need for packaging with bioactive compounds that neutralize infectious contaminants to reduce transmission risks. Essentially, with the likelihood that SARS-CoV-19 continues to move about the population, we want to avoid future outbreaks that consume health care infrastructures.

## Figures and Tables

**Table 1:** Micronutrients and their role in COVID-19.

Vitamins and Minerals	Food Sources	Actions	Role	Reference
Vitamin A	Meat, poultry, fish, dairy, eggs	Shows efficiency for immune function and resistance to infection	Immunomodulatory, Anti-inflammatory	[76]
Vitamin B1 (thiamine)	Meat, poultry, fish, whole-grains, brown rice dried beans, soybeans, nuts,	Eliminates the SARS-CoV-2 virus by triggering cell-mediated and antibody-mediated immunity	Supports immune response	[77]
Vitamin B2 (riboflavin)	Calf liver, fish, nuts, wild rice, dark green leafy vegetables, mushrooms, certain fruits and legumes, beer, yeast, milk, cheese, egg,	Reduces number of pathogens in the blood plasma of COVID-19 patients, and reduce the risk of transfusion–transmission of COVID-19.	Supports immune response	[77,78]
Vitamin B3 (niacin)	Meat, liver, beans	Reduces viral infection & stimulates defense mechanisms Reduces neutrophil infiltration in patients with ventilator-induced lung injury	Supports immune response	[77]
Vitamin B6 (pyridoxine)	Cereal grains, vegetables (carrots, spinach, peas), milk, potatoes, eggs, cheese, fish, liver, meat,	Relieves COVID-19 symptoms by improving immune response, supporting endothelial integrity, preventing hyper-coagulability & reducing pro-inflammatory cytokines	Supports immune response	[77]
Vitamin B12 (cyanocobalamin/cobalamin)	Meat, milk, egg, fish, and shellfish	Essential role in improved immune system function	Supports immune response	[77]
Vitamin C	Citrus fruits kiwi, tomato, pineapple, kale, spinach, beef liver, milk, cabbage, broccoli, chicken, oysters, strawberries	Reduces symptom duration Reduces mortality Prevents COVID-19 progression Decreases risk of respiratory failure requiring a ventilator Reduces death rate & dependency on ventilator	Antioxidant, immunomodulatory	[79]
Vitamin D	Wild mushroom, fungi, fortified, bread fortified orange juice, milk, eggs, cheese, yogurt, fortified margarine	Improves prognosis in older patients Prevents respiratory infections	Antioxidant, immunomodulatory	[80]
Vitamin E	Vegetable oils, Nuts, seeds, avocado, green leafy vegetables, mango, salmon fortified cereals	Antioxidant defense Role in immune response & to reduce viral pathogenicity	Antioxidant, immunomodulatory	[81]
Selenium	Whole grains, nuts, mushrooms, dairy products, poultry, cereals, red meat, seafood	Essential for protection against viral infection	Antioxidant, ROS balance in inflammation, immune-cell function	[79]
Zinc	Fortified breakfast cereal, nuts, beans, poultry, red meat, whole grains, crustaceans, mollusks	Reduces inflammatory reaction Increases ventilator-free days Organ failure-free days Acute inflammation-free days	Antioxidant, anti-inflammatory, reduces ROS in viral infection	[80]

**Table 2:** Biological activities of foods bioactive compounds.

Food Source	Compounds	Biological Activities	Reference
Onion	Quercetin, thiosulfinates, & anthocyanins	Antioxidant	[82]
Citrus fruits	Hesperidin	Antioxidant, anti-inflammatory, antiviral	[83]
Garlic	Diallyl disulphide, alliin, polyphenols, proteins	Antioxidant, antiviral	[84]
Honey	p-coumaric acid, ellagic acid	Antimicrobial, antiviral	[85]
Tea Plant	Gallic acid, theaflavin-3,3′-digallate, quercetin, catechins	Antioxidant, antiviral, immunomodulatory	[86]
Cranberry	Myricetin	Antiviral	[87]
Barberry	Berbamine, berberine	Anticancer	[82]
Turmeric	Curcumin	Anti-inflammatory	[83]
Soybean	Flavonoids, Isoflavones, phytosterols, saponins & organic acid	Antioxidant	[82]
Banana	Bananin	Antiviral	[88]
Long pepper, black pepper	Piperine	Anticancer	[82]
Plum	Anthocyanins, protocatechuic acid	Antioxidant	[82]
Grapes, berries	Quercetin	Antioxidant, anti-inflammatory	[89]
Kale	Kaempferol	Anti-inflammatory	[90]
Avocado, pistachio, almond	Β-sitosterol	Anti-inflammatory	[89]
Mango	Flavonoids, xanthones, phenolic acids, triterpenes	Antioxidant, antiviral	[82]
Nuts, seeds	Stigmasterol	Antiviral	[89]
Red grape	Resveratrol	Anti-inflammatory	[89]

**Table 3:** Dietary behavior in different countries on COVID-19.

	Colombia [119]	Poland [120]	Italy [100]	France [121]	Saudi Arabia [122]	Germany [123]	China [124]	Spain [125]
Ate more								
Increase	45%	-	-	-	-	40%	31%	64%
Decrease	20%	-	-	-	-	21%	17%	36%
As before	35%	-	-	-	-	39%%	52%	-
Weight gain								
Yes	22%	40%	37%	38%	38%	31%	-	13%
No	38%	10%	52%	18%	26%	16%	-	47%
Unknown	40%	50%	11%	44%	36%	53%	-	40%
Fast food								
Increase	21%	8%	-	-	-	-	-	5%
Decrease	34%	37%	-	-	-	-	-	35%
As before	45%	55%	-	-	-	-	-	60%
Snacking								
Increase	48%	30%	33%	24%	45%	-	25%	37%
Decrease	22%	10%	11%	18%	19%	-	37%	16%
As before	30%	60%	56%	58%	36%	-	38%	47%
Meals out of home								
Increase	-	-	-	-	-	7%	-	21%
Decrease	-	-	-	-	-	80%	-	50%
As before	-	-	-	-	-	13%	-	29%
Alcohol intake								
Increase	7%	18%	16%	12%	-	-	26%	11%
Decrease	18%	11%	13%	12%	-	-	39%	57%
As before	22%	71%	71%	39%	-	-	35%	32%
Never	53%	-	-	23%	-	-	-	-
Water intake								
Increase	36%	-	20%	-	57%	-	-	-
Decrease	26%	-	8%	-	7%	-	-	-
As before	38%	-	72%	-	36%	-	-	-
Physical activity								
Increase	23%	-	36%	26	27%	-	-	16%
Decrease	48%	-	11%	50%	52%	-	-	59%
As before	18%	-	16%	24%	21%	-	-	19%
Never	11%	-	37%	-	-	-	-	6%
Home cooking								
Increase	66%	-	-	-	73%	96%	65%	45%
Decrease	5%	-	-	-	4%	4%	9%	4%
As before	23%	-	-	-	23%	-	26%	51%
Never	6%	-	-	-	-	-	-	-

- Data not available. *N*, number of respondents: Colombia = 2745, Poland = 407, Italy = 2678, Saudi Arabia = 2255, Germany = 1964, China = 1994, Spain = 7514, France = 4005.

## Data Availability

The data presented in this study are available upon request from the corresponding author.
